# Supplementary material for: Complexome profiling of the Chlamydomonas psb28 mutant reveals TEF5 as an early PSII assembly factor
Source: Plant Cell. 2025 Mar 17;37(6):koaf055. doi: 10.1093/plcell/koaf055 (PMC12164590; doi:10.1093/plcell/koaf055)
Supplement: koaf055_Supplementary_Data [file koaf055_supplementary_data.zip › psb28_interactive.zip!/plots/intensity/qualityPlots/peptides/_Cre12.g525150.t1.2.html]

### Cre12.g525150.t1.2

Trivial name:   
SeqCoverage [median]: 27.60  
Mass: 69.26 kDa  
  
 

| Sequence | MissedCleavage | Mass [Da] | wtIntensity | muIntensity | Score |
| --- | --- | --- | --- | --- | --- |
| AAAEAAEAQVK | 0 | 1057.54 | 899.06 | 0.00 | 75.65 |
| AAANAAQEEVAAAR | 0 | 1341.66 | 804.98 | 874.70 | 126.19 |
| AAEELAAASSDSAK | 0 | 1319.62 | 573.42 | 574.88 | 107.03 |
| AALAEQLEAAAAER | 0 | 1412.73 | 271.60 | 1767.50 | 128.54 |
| AAQAAAAASIAAAAEQAEK | 0 | 1712.87 | 0.00 | 1531.58 | 35.04 |
| AATAAVEEAR | 0 | 987.50 | 1382.05 | 1347.14 | 85.73 |
| ALAAQLLAAR | 0 | 996.61 | 1052.97 | 3856.75 | 149.40 |
| ALNVSLAAASAEATAAR | 0 | 1585.84 | 0.00 | 904.29 | 63.18 |
| AQLDALQAEVEAQR | 0 | 1540.78 | 0.00 | 861.31 | 37.77 |
| DVVAQLADAQK | 0 | 1156.61 | 0.00 | 337.31 | 103.55 |
| DVVAQLADAQKELEHAK | 1 | 1863.97 | 0.00 | 927.23 | 53.40 |
| SAGGAPEAPTAPSASGK | 0 | 1454.70 | 316.48 | 620.64 | 117.79 |
| SGIEGLRQEMQMLQEER | 1 | 2032.97 | 0.00 | 773.36 | 44.57 |
| TAALAELTAVK | 0 | 1086.63 | 385.83 | 1505.08 | 92.24 |
|  |  |  | 5686.4 | 15881.8 |  |
